# Supplementary material for: Oral health inequality in Canada, the United States and United Kingdom
Source: PLoS One. 2022 May 4;17(5):e0268006. doi: 10.1371/journal.pone.0268006 (PMC9067688; doi:10.1371/journal.pone.0268006)
Supplement: S1 File — (PDF) [file pone.0268006.s001.pdf]

Contract number: 18-SSH-UTO-5761 (Chari)
Title: Magnitude and changes in socioeconomic inequalities in oral health over time in United Kingdom (UK), United States (US) & Canada.

MICRODATA RESEARCH CONTRACT

(Hereinafter referred to as the "Contract")

BETWEEN:

HER MAJESTY THE QUEEN IN RIGHT OF CANADA, as represented by the Minister responsible for Statistics Canada,

(Hereinafter referred to as "Statistics Canada"),

AND:

Malini Chari ; University of Toronto

Carlos R. Quinonez ; University of Toronto

(Hereinafter referred to as Researcher(s))

Each a "Party" and collectively referred to as "Parties".

Recitals

1. Statistics Canada requires the services of the Researcher(s) to perform Special Services of statistical research and analysis, as described herein, pursuant to the *Statistics Act* R.S.C. 1985 chapter S-19;

2. The performance of these Special Services requires that the Researcher(s) has/have access to the Information in Appendix D;

3. Subsection 5(3) of the *Statistics Act* provides that any persons retained under contract to perform Special Services for the Minister pursuant to the *Statistics Act*, and the employees and agents of those persons shall, for the purposes of the *Statistics Act*, be deemed to be employed under the *Statistics Act* while performing those services;

4. Subsection 6(1) of the *Statistics Act* provides that any person deemed to be employed pursuant to the *Statistics Act* shall, before entering on his/her duties, take and subscribe the oath or solemn affirmation contained in that subsection;

5. To perform these services and to have access to confidential information, the Researcher(s) must become Deemed Employee(s) of Statistics Canada, and is/are required to take the Oath of Secrecy and must adhere to Statistics Canada's security and confidentiality requirements;

6. Pursuant to section 13 of the Act, documents or records (Administrative Data) maintained in any department or in any municipal office, corporation, business or organization can be obtained and brought into Statistics Canada for the purpose of this Contract. All Administrative Data required for the purpose of this Contract must be listed in Appendix 'G';

7. Researchers can also gather and bring into Statistics Canada Premises, Publicly Available Information required for the purpose of this Contract;

8. The Proposed Output and all materials (excluding Other Source Data) brought into Statistics Canada premises (which includes Research Data Centres) by Researcher(s) pursuant to the execution of the Special Services will be subject to the *Access to Information Act,* R.S.C., 1985, c. A-1 and the *Privacy Act,* R.S.C., 1985, c. P-21;

9. Administrative Data brought into Statistics Canada premises by Researcher(s) pursuant to the execution of the Special Services will be subject to the confidentiality provisions of subsection 17(1) of the Act and subsection 24(1) of the Access to Information Act;

10. Statistics Canada wishes to establish the terms and conditions under which the Researcher(s) is/are retained to perform Special Services for the Minister pursuant to the *Statistics Act*, notably to ensure the appropriate use and the protection of the confidentiality of the Information to which the Researcher(s) may have access during the performance of these Special Services;

NOW THEREFORE the Parties agree as follows:

1. DEFINITIONS AND INTERPRETATIONS

1.1 Definitions

In this Contract, a capitalized term has the meaning given to it in this section, unless the context indicates otherwise:

“Administrative Data”
Administrative Data means information that is collected by other organizations and departments for their own purposes and is sought at the micro or aggregate level by Statistics Canada in respect to the objects of the Statistics Act. It excludes Publicly Available Information and data held by an individual. The Administrative Data can be brought onto Statistics Canada premises by Researcher(s) under section 13 of the Statistics Act for use in the performance of Special Services and listed in Appendix G.

“Administrative Data Provider”
Administrative Data Provider refers to the government department, corporation, business or organization from which the Researcher(s) receives the Administrative Data.

“Deemed Employee”
Deemed Employee means any person, not currently an employee of Statistics Canada, retained to perform Special Services for Statistics Canada pursuant to the *Statistics Act*, for which access to Information protected by the *Statistics Act* is required in order to perform the Special Services.

“Information”
Information means the confidential identifiable microdata provided to Researcher(s) by Statistics Canada and listed in Appendix D, pursuant to this Contract, and statistical aggregates thereof that could directly or indirectly identify a Person.

“Person”
Person means an individual, a corporation incorporated under any Act of Canada or a province or territory, a partnership, an association or an unincorporated business.

“Personal Identifiers”
Personal Identifiers refers to information that may lead to the direct identification of an individual.

“Proposed Output”
Proposed Output means output/work created by Deemed Employee(s) as a result

of providing Special Services outlined in Appendix C.

“Publicly Available Information”
Publicly Available Information means data obtained from the internet, as well as data obtained under licence with or without a fee, and can be made available to any member of the public under a licence agreement. Publicly Available Information is not brought into Statistics Canada under the Act.

“Special Services”
Refers to statement of work described in Appendix C.

“Statistics Canada Premises”
Statistics Canada Premises refers to a building or part of a building designated as Statistics Canada premises. This includes all Statistics Canada Research Data Centres.

1.2 <u>Interpretation of Appendices</u>

This Contract contains the following Appendices, which form an integral part of this contract:

(a) Appendix A – Security Requirements
(b) Appendix B – Operational Requirements
(c) Appendix C – Description of Special Services to be provided to Statistics Canada by Researcher(s)
(d) Appendix D – Information and related documentation provided to Researcher(s)
(e) Appendix E – Documents to be provided to Researcher(s)
(f) Appendix F – Conflict of Interest Declaration Form
(g) Appendix G – Administrative Data Metadata

In case of inconsistency or conflict between a provision contained in the part of the Contract preceding the signatures and a provision contained in any of the appendices, the provision contained in the part of the Contract preceding the signatures will prevail.

2. STATEMENT OF WORK

2.1 The Researcher(s) is/are hereby retained to perform the Special Services as documented in Appendix C, for the Minister pursuant to the *Statistics Act*.

2.2 Special Services shall consist of carrying out the research project and in providing the Proposed Output as described in Appendix C, in accordance with the requirements contained in this Contract.

3. INFORMATION ACCESSED BY DEEMED EMPLOYEE(S)

Subject to this Contract, Statistics Canada shall grant Deemed Employees access to the Information required to perform Special Services for Statistics Canada.

4. TERMS AND CONDITIONS OF ACCESS TO THE MICRODATA

4.1 Subject to the terms and conditions contained in this section and the Security Requirements in Appendix A as well as the Operational Requirements in Appendix B, Statistics Canada will provide access to the Information to the Researcher(s)

for the purpose of performing the Special Services.

4.2 Such access will be provided only to the extent necessary, at Statistics Canada’s discretion, for the performance of the Special Services.

4.3 Such access will only be provided on Statistics Canada premises and using equipment provided and/or specifically designated by Statistics Canada.

4.4 The Researcher(s) acknowledge and agree that, in order to have and maintain access to the Information, the Researcher(s) shall comply with the following pre-access requirements:

4.4.1 Having been granted at minimum, a “Reliability” security status as defined in the federal Policy on Government Security;

4.4.2 Having taken the oath of office, as required by section 6 of the *Statistics Act;*

4.4.3 Having read, understood and will comply with the relevant Statistics Canada policies, directives, guides and guidelines listed in Appendix E;

4.4.4 Having read, understood and will comply with the *Values and Ethics Code for the Public Sector*, Statistics Canada Code of Conduct and the Policy on Conflict of Interest and Post-Employment listed in Appendix E;

4.4.5 Having declared in Appendix C:

4.4.5.1 That the sole purpose of the research project is statistical research,

4.4.5.2 The sources of monetary or in kind support they are receiving to carry out the Research Project;

4.4.6 The Researcher(s) understand the potential penalties should they contravene the terms and conditions of access to the Information and the penalties should the Researcher(s) contravene the *Statistics Act* and any applicable related Acts, including the *Income Tax Act* or the *Excise Tax Act*.

5. LIMITATIONS ON USE OF INFORMATION

5.1 The Researcher(s), in the course of carrying out this Contract, may not use any of the information gained by accessing the Information for any other purpose except that which was agreed upon in this Contract.

5.2 Access to the Information is being provided for the statistical and research purpose outlined in the Statement of Work in Appendix C.

5.3 The Researcher(s) shall not disclose any of the Information to anyone other than current Statistics Canada employees involved in the review or evaluation of any aspect of the research project or to other Deemed Employees who have been approved for the same Special Services and therefore are also authorized to have access to the same Information.

5.4 The Researcher(s) shall ensure that no attempts are made to link the Information supplied herein to any other files in order to relate the particulars to any identifiable Person.

6. PENALTIES

As Deemed Employees of Statistics Canada, and having taken the oath/solemn affirmation of secrecy set out in section 6 of the *Statistics Act*, Researcher(s):

6.1 Remains/Remain subject to the oath/solemn affirmation of secrecy even after the term of the Contract has ended.

6.2 Is/are subject to all the applicable penalties provided for in the *Statistics Act* for contravention of any of the confidentiality provisions and are liable on summary conviction to any of the applicable fines or imprisonment terms.

6.3 Is/are prohibited from disclosing information related to any Person (subsection 17(1) of the *Statistics Act*) obtained under the *Statistics Act.* Researcher(s) contravening subsection 17(1) of the *Statistics Act* is/are guilty of an offence and liable on summary conviction to a fine not exceeding one thousand dollars or to imprisonment for a term not exceeding six months or to both (paragraph 30(c) of the *Statistics Act*).

6.4 Is/are prohibited from disclosing confidential information obtained through the course of their employment that might exert an influence on or affect the market value of any stocks, bonds or other security or any product or article, or using the same information to speculate in any stocks, bonds or other security or any product or article (section 34 of the *Statistics* Act). Researcher(s) contravening section 34 of the *Statistics Act* is/are guilty of an offence and liable on summary conviction to a fine not exceeding five thousand dollars or to imprisonment for a term not exceeding five years or to both.

6.5 Is/are reminded that if they are accessing data from sources other than Statistics Canada, in accordance to this Contract, then they are subject to all the applicable penalties provided for in related and applicable laws for contravention of any of the confidentiality provisions and are liable on summary conviction to any of the applicable fines or imprisonment terms.

7. OWNERSHIP AND COPYRIGHT OF INFORMATION

7.1 Statistics Canada is the owner and/or steward of the Information and related documentation listed in Appendix D and Parties agree that this Contract pertains to the use of the Information and related documentation to produce the Proposed Output for Statistics Canada. Nothing contained herein shall be deemed to convey any title or ownership interest in the Information or the related documentation to the Researcher(s).

7.2 Statistics Canada is also the steward of Administrative Data and all associated documentation, as well as Publicly Available Information brought onto Statistics Canada Premises by Researcher(s).

7.3 Copyright in the Proposed Output shall vest in Her Majesty the Queen in Right of Canada. The Researcher(s) may be required to provide to Statistics Canada, at the completion of the Contract, or at such other time as Statistics Canada may require; a written permanent waiver of Moral rights from every author who contributed to the Proposed Output.

7.3 Copyright in any subsequent work created by the Researcher(s) using the Proposed Output shall vest in the Researcher(s).

8. USE OF AND PUBLISHING OF PROPOSED OUTPUT

8.1 Release of the Proposed Output by Statistics Canada may be considered by Statistics Canada in consultation with the Principal Researcher.

8.2 Statistics Canada reserves the right:

8.2.1 To publish in whole or in part or an amended/derived version of the Proposed Output; or

8.2.2 Not publish at all, any part of the Proposed Output

8.3 Use of the Proposed Output by Researcher(s) will be governed by the Statistics Canada Open License Agreement which can be found at the link below. This license agreement allows Researcher(s) to use Statistics Canada information without restrictions on sharing and redistribution, for commercial and non-commercial purposes.

http://www.statcan.gc.ca/eng/reference/licence-eng

9. CONFLICT OF INTEREST

9.1 Researcher(s) engaged as Deemed Employee(s) in the course of carrying out this Contract shall conduct themselves in accordance with the principles and spirit of the *Values and Ethics Code for the Public Sector*, Code of Conduct at Statistics Canada and the Policy on Conflict of Interest and Post-Employment found in Appendix E.

9.2 Researcher(s) must complete the Conflict of Interest Declaration Form found in Appendix F.

9.3 If Researcher has a conflict, the Researcher must fill out a Confidential Report to be provided by the Statistics Canada representative. This Report must be approved by the Director General, Human Resources Branch, Statistics Canada, who may require corrective action prior to providing the approval.

10. DESIGNATED REPRESENTATIVES

10.1 Any notice to be given to Statistics Canada pursuant to this Contract will be addressed to:

Director
Microdata Access Division
Statistics Canada
9R, R.H. Coats Building
Ottawa, ON K1A 0T6

10.2 And any notice to be given to the Researcher(s) will be addressed to:

Malini Chari
University of Toronto
124 Edward Street, Toronto, ON, M5G 1G6, CAN
(647)916-7568

11. PAYMENT

Funding arrangements and payment modalities for purposes of this Contract are outlined in a separate Letter of Agreement between Statistics Canada and the Researcher(s).

12. TERM

This Contract comes into force when signed by all Parties, beginning on the date of the later signature, and shall continue until 2021-08-31 (YYYY-MM-DD) unless terminated earlier in accordance with section 13.

13. TERMINATION

13.1 This Contract may be terminated for any reason by either Party upon thirty (30) day Notice of termination having been made in writing to the other Party, or at a time otherwise agreed upon by the Parties. Such termination will take effect on the expiry of the notice period.

13.2 Statistics Canada will terminate this contract immediately upon giving written notice to the Researcher(s) where the Researcher(s) commits or permits a breach of any of the terms and conditions contained in this Contract.

14. NOTICE OF CHANGE

Researcher(s) shall inform Statistics Canada, in writing, within thirty (30) days of any changes in their programs and policies, as well as of any legislation or regulation that may affect this contract.

15. AMENDMENT

No amendment to this Contract will be effective unless it is made in writing and signed by the persons occupying the positions of the signatories of this Contract.

16. GENERAL

16.1 No Assignment
The Researcher(s) acknowledges that this Contract will not be assigned in whole or in part without the prior written consent of Statistics Canada, and any assignment made without such consent will be void and of no effect.

16.2 Notices
Unless otherwise specified in the Contract, where in this Contract any notice or other communication is required to be given or made by either Party, it will be in writing and be effective if sent by registered mail, e-mail, facsimile, postage prepayment or delivered in person, addressed to the respective Party at the contact information outlined under Section 10 of this Contract. Any notice or other communication will be deemed to have been given: if by registered mail when the postal receipt is acknowledged by the other Party; if by e-mail or facsimile on the day after the e-mail or facsimile was sent; if by mail on the eighth (8th) calendar day following the day of mailing.

16.3 Survival
The sections of this Contract regarding restrictions on use, confidentiality, conflict of interest, offenses and punishment, disclaimer of warranty, termination and general, and any other provisions which by their nature survive the termination or expiry of this Contract, will survive any termination or expiration of this Contract.

16.4 Law

This Contract shall be governed by and construed in accordance with the laws of the Province of Ontario and all applicable laws of Canada.

16.5 Entire Agreement
The Contract constitutes the entire agreement between Parties with respect to the subject matter described herein and supersedes all previous negotiations, communications and other agreements on the same topic, unless specifically incorporated by reference in this Contract.

16.6 Waiver
Any tolerance or indulgence demonstrated by one Party to the other, or any partial or limited exercise of rights conferred on a Party, shall not constitute a waiver of rights, unless expressly waived in writing by that Party.

16.7 Severance (Delete 16.7 if Researcher(s) is/are federal employee(s))
If any provision of this Contract, whether in whole or in part, is held by a court of competent jurisdiction to be void or unenforceable, such provision or portion thereof declared invalid or unenforceable shall be deemed to be severable and shall be deleted from this Agreement and all remaining terms and conditions of this Contract will continue to be valid and enforceable.

Contract number: 18-SSH-UTO-5761 (Chari)
Title: Magnitude and changes in socioeconomic inequalities in oral health over time in United Kingdom (UK), United States (US) & Canada.

IN WITNESS WHEREOF, this Contract has been executed on behalf of:

FOR STATISTICS CANADA:

___________________________________	____
Director, Microdata Access Division	Print Name
___________________________________	____
Witness	Print Name

DATED at Ottawa, Province of Ontario, this _________ day of _________ (month) _________ (year).

Contract number: 18-SSH-UTO-5761 (Chari)
Title: Magnitude and changes in socioeconomic inequalities in oral health over time in United Kingdom (UK), United States (US) & Canada.

FOR THE PRINCIPAL RESEARCHER AND CO-RESEARCHER(S):

____________________________________	Malini Chari
Principal Researcher (sign here)	Print Name
____________________________________	____________________________________
Witness (sign here)	Print Name

DATED at Toronto, Province of Ontario, this _____ day of _______ (month) 2018.

Carlos R. Quinonez	____________________________________
Co- Researcher (sign here)	Print Name
____________________________________	____________________________________
Witness (sign here)	Print Name

DATED at Toronto, Province of Ontario, this _____ day of _______ (month) 2018.

(Complete for all deemed employees signing contract)

Contract number: 18-SSH-UTO-5761 (Chari)
Title: Magnitude and changes in socioeconomic inequalities in oral health over time in United Kingdom (UK), United States (US) & Canada.

APPENDIX A
SECURITY REQUIREMENTS

The Information is designated as confidential*.* The security requirements described below are the minimum requirements that Researchers must comply with.

1. The Researcher(s) shall not remove any of the Information or any confidential sensitive statistical information provided pursuant to this contract from Statistics Canada Premises.

2. The Researcher(s) may request the removal of information subject to the following conditions.

   a) Any material to be removed from the Statistics Canada premises by the Researcher(s) must first be screened by Statistics Canada to ensure that there is no risk of disclosure of confidential information, which includes any information that may lead to the identification of a Person as defined in section 17 of the *Statistics Act*.

3. Researcher(s) must take all precautions to avoid disclosure of confidential information.

4. The Researcher(s) must use only equipment that is provided in the secure Statistics Canada Premises. Such equipment must never be removed from the Statistics Canada Premises.

5. The Researcher(s) shall make no attempt to tamper with the configuration or security features of any computer workstation they are provided with to perform the Special Services.

6. The Researcher(s) shall not attempt to compromise the security of the computing environment. Without limiting the generality of the foregoing, this includes using screen capture/sharing software or devices, and allowing unauthorized individuals to view the data.

7. Should the Researcher(s) become aware of a real or suspected breach of security, an unauthorized disclosure or unauthorized access of confidential data, they must inform the Statistics Canada Representative without delay.

8. **Network Use**
   In accordance to Statistics Canada IT Security Policies as defined in the Network Use Policy Researcher(s) acknowledge that the following limitations apply to all use of the Research Data Centre wide area network:

   Researchers must not conduct any unlawful or unacceptable activity. Without limiting the generality of the foregoing this includes:

   - **Attempting to defeat information technology security features**, through such means as using anti-security programs; using someone else’s password, user-identification or computer account; disclosing one’s password, network configuration information or access codes to others; or disabling anti-virus programs. (Government Security Policy).

   - **Destroying, altering or encrypting data** without authorization and with the intent of making it inaccessible to others with a lawful need to access it.

APPENDIX B
OPERATIONAL REQUIREMENTS

1. The Statistics Canada representative shall provide Researcher(s), with copies of all relevant Statistics Canada policies related to confidentiality, privacy and security and standard operating procedures listed in Appendix E.

2. Should a conflict of interest be acquired or develop during the life of this Contract, the Researcher(s) shall inform the Statistics Canada representative without delay. It may be necessary to submit or modify a Confidential Report. The performance of the Special Services will be suspended until the Confidential Report is approved by the Director General, Human Resources Branch, Statistics Canada, who may request corrective action prior to approval.

3. The Researcher(s) shall provide Statistics Canada with the programs, the supporting documentation and any other information necessary to reproduce all the tabulations and analytical output they would like to remove from Statistics Canada premises under Appendix A (1)(2).

APPENDIX C

DESCRIPTION OF SPECIAL SERVICES TO BE PROVIDED TO STATISTICS CANADA BY RESEARCHER(S)

DESCRIPTION OF STATEMENT OF WORK
See attached proposal entitled Magnitude and changes in socioeconomic inequalities in oral health over time in United Kingdom (UK), United States (US) & Canada.

DESCRIPTION OF PROPOSED OUTPUT

- Vetted Statistical output associated with the Statement of Work;

LOCATION OF WORK

Contact Name	RDC
Malini Chari	UTO - University of Toronto
Carlos R. Quinonez	UTO - University of Toronto

COMPLETION DATE

See Section 12. 2021-08-31

SOURCE OF FUNDING

None

Contract number: 18-SSH-UTO-5761 (Chari)
Title: Magnitude and changes in socioeconomic inequalities in oral health over time in United Kingdom (UK), United States (US) & Canada.

APPENDIX D
INFORMATION AND RELATED DOCUMENTATION PROVIDED TO RESEARCHER(S)

Product Description
5071_CHMS-ECMS_C1-2007-2009

APPENDIX E
DOCUMENTS TO BE PROVIDED TO RESEARCHER(S)

The following documents will be provided to Researcher(s).

- Research Data Centre Researcher Guide
- Research Data Centre Orientation Session
- Code of Conduct at Statistics Canada
- *Values and Ethics Code for the Public Sector*
- Policy on Conflict of Interest and Post-Employment

Contract number: 18-SSH-UTO-5761 (Chari)
Title: Magnitude and changes in socioeconomic inequalities in oral health over time in United Kingdom (UK), United States (US) & Canada.

APPENDIX F
CONFLICT OF INTEREST DECLARATION FORM

Deemed Employees are required to conduct themselves in accordance with the *Values and Ethics Code for the Public Sector*, including taking all possible steps to prevent and resolve any real, apparent or potential conflicts of interests between their official responsibilities and their private affairs in favour of the public interest.

As a Deemed Employee of Statistics Canada, I acknowledge that I have read the *Values and Ethics Code for the Public Sector*, that I will not undertake any projects in the future that would benefit from my access to any confidential data as a result of this contract, and I further declare that I will comply with the code and that:

__I have no conflicts to declare, or

__I may have a conflict and will complete the "Confidential Report" as required.

____________________________	Malini Chari	________________
Signature of Deemed Employee	Printed Name of Deemed Employee	Date

__I have no conflicts to declare, or

__I may have a conflict and will complete the "Confidential Report" as required.

____________________________	Carlos R. Quinonez	________________
Signature of Deemed Employee	Printed Name of Deemed Employee	Date

(Complete for all deemed employees signing contract)

APPENDIX G
ADMINISTRATIVE DATA METADATA

DESCRIPTION OF ADMINISTRATIVE DATA
Copy and complete for each administrative dataset.
Administrative Data Set #1:

**Name of Administrative Data Provider:**	
**Source of Administrative Data:**	
**Type of Administrative Data Provider:** (private sector, Federal, Provincial/Territorial or Municipal government)	
**Type of Permission granted to use Administrative Data** (Example -If Administrative Data are from the Researcher(s)' employer, then the Principal Researcher must provide a copy of an approval letter from: an Ethics Review Board in the case of a university or the private sector; share agreement; or from the deputy head in the case of a public sector. Attach documents as required)	
**Themes of the Administrative Data:**	
**Description of Contents:** (year of collection, key variables or refer to proposal if sufficient)	
**Record Layout or Administrative Data Dictionary:** (if yes, name files)	
**Quality Assessment of Administrative Data Completed by Data Provider (y/n) :**	
**Frequency of Reception**: (one-time, monthly, yearly)	
**Method of Reception:** (email, USB, other)	
**Personal Identifiers Included (y/n) :** (if yes, the identifiers will be removed before use in the RDCs.)	
**Description of linkage, merge or pooling of Administrative Data with Statistics Canada data** (Option to reference the proposal if the description is sufficient)	
**Administrative Data Custodian:**	**Division: MAD**<br>**FRC#: 84100**<br>**PE#: 6587**

For information - Conditions of use of Administrative Data If used solely for Special Services described above:

- Must be destroyed by Statistics Canada after completion of Special Services in accordance with the Statistics Canada Directive on the Management of Statistical Microdata Files.
- If Administrative Data are to be used for other Statistics Canada projects: Terms of use shall be negotiated in a separate agreement.
