# Supplementary material for: Oral health inequality in Canada, the United States and United Kingdom
Source: PLoS One. 2022 May 4;17(5):e0268006. doi: 10.1371/journal.pone.0268006 (PMC9067688; doi:10.1371/journal.pone.0268006)
Supplement: Attachment — Submitted filename: Response to Reviewers.docx [file pone.0268006.s004.docx]

**Reviewer #1**

**Comments:**

The definition of outcome variables is not clear. The authors have not provided the precious detail about how to dichotomized “untreated decayed teeth”,” filled teeth”, and “edentulism”. The authors need to clarify how exactly the initial data separated into two possible values, maybe 0 and 1.

**Response:**

Please find below the categorization of outcome variables used in the analysis. This has been added under “Outcome Variables” on page 8.

| Table 2: Summary and description of variables used in the analysis | | | | |
| --- | --- | --- | --- | --- |
| **Variable** | | **Code** | | **Label** |
| **Outcome Variables** | **Untreated Decay** | 0 | No decayed teeth present | No decay |
|  |  | 1 | ≥1 Decayed teeth present | Decay present |
|  | **Filled teeth** | 0 | No filled teeth | No fillings |
|  |  | 1 | ≥1 filled teeth present | Fillings present |
|  | **Edentulism** | 0 | 0-31 missing teeth | Dentate |
|  |  | 1 | 0-32 missing teeth | Edentate/Edentulous |

**Comments:**

Now it is not possible to correctly interpret the estimations for SI & RII. For example, consider the “untreated decayed teeth” as the first indicator. If the dichotomous variable defined 0 for those had not any untreated decayed teeth, and 1 for the others, this variable would be an adverse outcome. As a result, positive SIs and RII greater than 1 indicates that untreated decayed teeth concentrated more among the rich. In the other word, the rich needs more help to satisfy needs for oral health. The interpretation of positive SIs and RII greater than 1 would be completely opposite if the If the dichotomous variable defined 1 for those had not any untreated decayed teeth, and 0 for the others. Now the authors just mentioned the words: “≥1 untreated decayed teeth (UD)”, and “≥1 filled teeth (FT)”. The dichotomizing approach for edentulism is also unclear.

**Response:**

The interpretation of SII and RII is associated with the hierarchical ranking of the socioeconomic group. In this analysis, the socioeconomic group was ranked from highest to lowest, highlighted on page 8, paragraph 2. Therefore, a positive SII denotes an increase in the outcome as one moves from the highest socioeconomic group to the lowest socioeconomic group. Similarly, a negative SII denotes a reduction in the outcome as we move from the highest to the lowest socioeconomic group. Therefore, an SII of 28.2 for untreated decay in the US means that the level of untreated decay increases by 28.2% as the socioeconomic status decreases, while an SII of -17.7 for filled teeth means that the level of fillings decreases by 17% as the socioeconomic status falls.

In terms of the RII which is interpreted as a ratio, the reference group is the highest socioeconomic group. Therefore, an RII of 4.7 for untreated decay in the US would mean that for every rich person with untreated decay, there are almost 5 poor people with untreated decay. Similarly, for filled teeth, it can be interpreted that for every 100 rich people with filled teeth, there are 81 people with filled teeth among the poor in US.

This means that a positive SII value and an RII greater than 1 indicate inequality favouring the rich, as highlighted under the analysis section on page 12, lines 267-268, and is indicative of higher oral health needs among the poor.

**Comments:**

Existing literate on health inequality monitoring, different complex measures of inequality including both absolute and relative measures provided. Some of these measures have been used more in available publications such as Gini index and Concentration index (). It would be useful that authors explain why they decided to calculate oral health inequality based on two selected measures.

**Response:**

Although the Gini coefficient has been used to measure socioeconomic inequality in health, it does not take into account the hierarchical nature of the socioeconomic variable and reports mainly on the inter-individual inequality^1^. While the Concentration Index overcomes the limitation of the Gini coefficient, it measures only relatively inequality and is similar to the RII^1^. In our study we wanted to measure both absolute and relative inequality as they provide different information about the same population, thus our choice of the SII and RII. Moreover, the SII and RII have not been used to compare oral health inequality between Canada, the United States and United Kingdom. This has been highlighted under “Analysis” on page 11, lines 242-246.

**Comments:**

The sample of the study included three surveys conducted before 2010 which seem to be old. The authors needs to explain the reasons or limitations they had for choosing old datasets.

**Response:**

The surveys chosen for this paper were earlier than 2010 as more recent national level data were not available for either Canada or the UK. The last nation-wide survey data available for Canada was the Canadian Health Measures Survey 2007-2009 and the Adult Dental Health Survey 2009 for the UK. This has been added to the manuscript under “Data Sources” on page 6, lines 156-157.

**Comments:**

The authors mentioned that edentulism was clinically verified in both the CHMS and NHANES but was self-reported in ADHS. It is strongly suggested that authors mentioned the related limitation in the interpretation of results in the “Discussion”.

**Response:**

This has now been addressed under “Discussion” on page 18&19, lines 385-387.

**Comments:**

The reason for selection of three countries of Canada, The US, and the UK needs more clarification. There are a couple of developed countries with the similar sociopolitical context, and various oral healthcare systems which have the chance included in the sample study of this paper.

**Response:**

This study was focused on comparing inequality between Canada, the US and UK, which is a common comparison in the health policy literature. All three countries are also liberal welfare states, each with unique health care systems in relation to each other, resulting in a good comparative context. Specifically, the US has a predominantly a privately organized, financed and delivered oral and general health care system, while Canada and the UK have national systems of universal health insurance covering hospital and physician care, yet Canada excludes oral health care and the UK does not. Finally, while there are other countries with similar socio-political contexts, there have been no previous studies comparing oral health inequality within and between Canada, the US and UK. This has been clarified in the introduction on page 4, lines 100-116.

**Reviewer #2:**

**Comments:**

General - I suspect that this manuscript was prepared at the same time as your J Public Health Dent and that this current manuscript now needs updating to cite your newly published research.

**Response:**

Thank you, this has been addressed in the Introduction section on page 5.

**Comments:**

Abstract - Please review the first sentence - starting 'there is little evidence' is not the strongest start to draw in the reader. And you present evidence in your introduction that there are at least two other papers in addition to your newly published paper on this subject.

**Response:**

This has been addressed in the abstract. On page 2, lines 51-54.

**Comments:**

Introduction - Towards the end of the introduction, there is a brief sentence telling us that there is other research comparing oral health inequality between Canada and the US (with references 16 and 17 cited). The reader wants to know what those papers (and your newly published one) have already told us about this subject - not just that they exist.

**Response:**

This has been addressed in the Introduction section on page 5, paragraph 1, lines 128-133.

**Comments:**

Table 1 - Expenditure: Please ensure that you present figures in the same format (ie to either one or two decimal places). Also delete the words 'predominantly private financing and public/private mixed model' - these are superfluous - the figures show the nature of the private/public mix in each country.

**Response:**

This has been addressed in table 1.

**Comments:**

Table 1 - Population covered: Needs a foot note with the source of these figures. Please also review the statement that 100% of UK public is covered by publicly provided dental services. Only around 50% of the UK population access NHS dentistry each year, which may be important to your discussion section when exploring why UK has the worst rate of untreated decay. Also consider including a figure for 'no coverage' in the UK.

**Response:**

Thank you for your comments, they are well taken. We have added the footnote. Also, from our understanding, in the UK, the NHS provides comprehensive oral health care, funded largely from taxation, but also supplemented by patient copayments for care provided by the General Dental Service, delivered through Primary Care Trusts. Children under 18 years, pregnant women, nursing mothers and individuals on welfare assistance are however entitled to treatment with no costs at point of care. The rest of the population receives dental care at subsidized rates, regulated by the national framework categories. Other than this, dentistry is also funded through private insurance. Overall, due to an expanding private practice market in the UK, patients contribute 54% toward oral health spending, while the government share is 46%. In other words, while the government contributes to only 46% of the oral health expenditure, 100% of the population is covered by some form of public insurance. This has been indicated in the table^2,3.^

Regarding an explanation for having the worst rate of untreated decay in the UK, this was addressed in the discussion considering the larger socio-political determinants of health, on page 20, 2^nd^ paragraph, lines 423-433.

**Comments:**

Outcome variables: Needs the term DMFT explained early in the section. Please clarify (for readers who may not be dental professionals) that edentulism means all teeth are missing.

**Response:**

DMFT: This has been addressed under the outcome variables section on page 7, paragraph 2, line 178-179. Edentulism: This has been addressed on page 8, paragraph 1, lines 188-189.

**Comments:**

Analysis - early on in this section, you need to state that only data relating to adults (aged over 16 years) was included in the analysis - it currently appears rather late in this section. You might even wish to state this in the data sources section.

**Response:**

This has been addressed in the introduction section on page 5, line 140, as well as under data sources on page 7, lines 173-174.

**Comments:**

SII and RII should have a reference to assist the reader who wants to understand more about this type of analysis

**Response:**

An additional reference has been added to the previously referenced to facilitate understanding of these indices^1,4,5^.

**Comments:**

Why and how did you age-standardise and sex-standardise the results - it wasn't clear why this was undertaken and how it aids interpretation of the data.

**Response:**

Age standardization was done to overcome the differences in the age composition in the three different samples. Direct age standardization was carried out as recommended by both the WHO and Statistics Canada^6,7^. For this purpose, either one population is mathematically adjusted to have the same age structure of the other population or both populations maybe mathematically adjusted to have the same age structure as a third population, also referred to as the standard population. In the case of this paper, all three samples were adjusted to the standard population of US 2000 Census. In this way all three samples were given the same age distribution structure. The results therefore account for the differences in age composition across time and countries. This has been added under the analysis section on page 12, second paragraph, lines 275-280. As for sex, this variable was not standardized but adjusted for during the regression analysis.

**Comments:**

Results - Table 4 The format of the title used in Table 2 of your J Public Health Dent paper is clearer. Also, the foot notes a and c do not seems to have a reference in the table itself

**Response:**

To clarify, before revision, Table 3 in the current paper is the equivalent of Table 2 in the J Public Health Dent paper, while Table 4 in the current paper was represented as Figure 1 in the J Public Health Dent paper.

However, after revisions, Table 4 represents sample characteristics and Table 5 represents the age-standardized prevalence of oral health outcomes by socioeconomic position (represented as Figure 1 in the J Public Health Dent paper).

The footnotes have been addressed.

**Comments:**

Table 5 - needs meaningful titles needed SII and RII need to be replaced by terms which can be understood by the reader without reference to the main text

**Response:**

This has been addressed. SII and RII have been replaced by Absolute Inequality and Relative Inequality, respectively. (Table 5 is now Table 6, after revisions)

**Comments:**

Discussion - sentence 2 - where has the term 'average level of oral health' been defined and explore in the results? This would be clearer couched as 'Untreated decay was worst in the UK........etc.'

**Response:**

This has been addressed as oral disease (UD and edentulism), under discussion on page 17, lines 357-358.

**Comments:**

Do your data provide evidence that the publicly funded care provided in UK is of a poorer standard of care than is available to US and Canadians? Or is there a difference of philosophy in relation to minimum-intervention dentistry in the UK compared to the other nations eg enamel-only caries is treated more conservatively rather than with fillings?

**Response:**

Our research does not provide any evidence on the quality of dental care in the three respective countries. Also, we do not provide any information on the type of interventions or philosophy behind the services provided. Our analysis provides potential explanations for inequality in the three countries, which may be related to the model of oral health care funding and delivery as well as larger socio-political context.

**Comments:**

Why refer to no cost treatment for children in the UK when the paper is about adult oral healthcare? Are you suggesting that better care as a child has an impact on adult oral health?

**Response:**

In this context, we are simply referring to the population coverage of public dental services in the UK, which also includes children. While there is research suggesting that better oral care in childhood can lead to better outcomes in adulthood, that is not what is being suggested in this context, rather a comparison of the extent and availability of public dental services, that might potentially explain our findings. This has been clarified on page 18, lines 365-367.

**Comments:**

Line 370 - where did Australia and New Zealand suddenly come from? Needs new references if

you are leaving this sentence in the manuscript.

**Response:**

Thank you for your comments. This is in reference to the study by Meija et al., in the introduction. We have clarified this in the introduction on page 4, lines 118-119.

**Comments:**

Line 377-387 - as highlighted in the first and last paragraphs of your discussion section, this is one of the most important findings of your study and should appear much earlier in the discussion section.

**Response:**

Thank you for your comments. This has been addresses in the first paragraph of the discussion on page 17, line 357.

**References**

1. Mackenbach JP, Kunst AE. Measuring the magnitude of socio-economic inequalities in health: an overview of available measures illustrated with two examples from Europe. Soc Sci Med. 1997 Mar;44(6):757–71.
2. Vujicic, M., Bernabé, E., Neumann, D., Quiñonez, C., Mertz, E. Health determinants and outcomes: Dental Care. In: Scheffler R, editor. World scientific handbook of global health economics and public policy: Volume 2. World Scientific Publishing Co. Pte. Ltd; 2016
3. Boyle S. United Kingdom (England): Health system review. Health Syst Transit. 2011;13(1):1-483, xix-xx.
4. World Health Organisation. Handbook on health inequality monitoring: with a special focus on low- and middle-income countries.Geneva: WHO Press; 2013.
5. Cheng NF, Han PZ, Gansky SA. Methods and software for estimating health disparities: The case of children’s oral health. Am J Epidemiol. 2008;168(8):906–14.
6. Ahmad OB, Boschi-Pinto C, Lopez AD, Murray CJ, Lozano R, Inoue M. Age Standardization rates: A new WHO standard [Internet]. World Health Organisation; 2001. (GPE Discussion Paper Series: No.31). Available from: <https://www.who.int/healthinfo/paper31.pdf>
7. Statistics Canada. Age-standardized Rates [Internet]. 2017. (The Daily). Available from: <https://www.statcan.gc.ca/en/dai/btd/asr>
